# Developing a European network of analytical laboratories and government institutions to prevent poisoning of raptors

**DOI:** 10.1007/s10661-021-09719-2

**Published:** 2022-01-20

**Authors:** Irene Valverde, Silvia Espín, Pilar Gómez-Ramírez, Pablo Sánchez-Virosta, Antonio J. García-Fernández, Philippe Berny

**Affiliations:** 1 Toxicology and Forensic Veterinary Service, Faculty of Veterinary, University of Murcia, Campus de Espinardo, Murcia, Spain; 2 College of Veterinary Medicine—Toxicology, Marcy L’Etoile, 1 av Bourgelat, 69280 Lyon, France

**Keywords:** European network, Laboratories, Forensic veterinary, Wildlife toxicology, Poison, Birds of prey

## Abstract

Many cases of wildlife poisoning in Europe have been reported causing population declines, especially in raptors. Toxicovigilance and risk assessment studies are essential to reinforce the knowledge of the number of illegal poisoning cases and the substances involved in these crimes. Many researchers and projects in different institutions have suggested the creation of a network to improve communication and share information between European countries. This article presents the results of the Short-Term Scientific Mission titled “Developing a Network of Analytical Labs and Government Institutions” supported by the COST Action *European Raptor Biomonitoring Facility* (CA16224), which aims to initiate a network of veterinary forensic toxicology laboratories, in order to improve communication among laboratories to prevent wildlife poisoning, especially in raptors. For this purpose, a questionnaire was designed and sent by email to 119 laboratories in Europe. It contained 39 questions on different topics (e.g. laboratory activities, analytical information). A total of 29 responses were received. Most participant laboratories work on veterinary forensic toxicology research and external cases at the same time, which provides a robust overview of the actual situation in the field. Analytical techniques and data collection methods should be harmonised, and communication between laboratories is encouraged to create a more effective network. The present study established contact between laboratories as an initial step to create a European network and compiled basic data to identify strengths and weaknesses that will help harmonise methodologies across Europe and increase pan-European capacities.

**Supplementary Information:**

The online version contains supplementary material available at 10.1007/s10661-021-09719-2.

## Introduction

In Europe, the use of poisons to kill wildlife and domestic animals is strictly prohibited by various regulations and directives (e.g. Directive 92/43 1992; Directive, 2009/147/EC 2010). According to the Directive 2008/99/EC (2008) on the protection of the environment, “killing, destruction, possession or taking of specimens of protected wild fauna or flora species” constitutes a criminal offence. Nevertheless, many cases of illegal animal poisoning have been reported in Europe (Guitart et al., 2010; Hernández & Margalida, 2008; Ntemiri et al., 2018; Parvanov et al., 2018).

Animal poisoning may occur due to different causes: (i) misuse of a registered chemical product or pesticide, (ii) abuse or illegal poisoning, when a chemical product, authorized or not, is used intentionally to kill animals using baits, (iii) as a result of a secondary poisoning (i.e. an animal scavenges or predates another animal already poisoned), or (iv) as an incidental case with a substance with an approved use (Berny, 2007; Hunter et al., 2005; Krone et al., 2017; Lambert et al., 2007). It has also been shown that illegal poisoning of raptors may result in population declines. Red kites (*Milvus milvus*) in Spain are a good example of such a situation (Mateo-Tomás et al., 2020).

Conflicts between humans and wildlife are the main reason why poison is used to kill animals (Berny, 2007; Bodega Zugasti, 2014; Mateo-Tomás et al., 2012) and should be the first step to deal with illegal wildlife poisoning. Due to the high incidence of animal poisoning, more restrictive and effective laws are urged by many as crucial measures to control this illegal activities (Bille et al., 2016; Hernández & Margalida, 2008; Mateo, 2010; Parvanov et al., 2018). Additionally, these laws must be enforced correctly, including training on environmental laws for public prosecutors, judges, lawyers and land users. Material and human resources for the prosecution for such crimes must be appropriately allocated by the authorities (Ntemiri et al., 2018; Ruiz-Suárez et al., 2015; Silva et al., 2018).

Despite existing laws, it has been demonstrated that banning of a product does not prevent it to be used to poison animals. However, the origin of these products is unclear, although old stocks, chemists or veterinarians could be the suppliers (Martínez-Haro et al., 2008). In addition, legally available products such as anticoagulant rodenticides (ARs) cause most of the acute poisonings in predators, probably because they are easily available at the supermarkets and widely used (Berny et al., 2010; Mateo, 2010). This scenario leads researchers to focus on product regulation, distribution and professional use and also on the control of banned chemical stocks (Martínez-Haro et al., 2008; Ruiz-Suárez et al., 2015). The products most frequently used in illegal baits are those with a low lethal dose (i.e. classified as acute toxicity 1 or 2 according to Classification, Labelling and Packaging (CLP) European Regulation, Regulation (EC) No. 1272/2008, 2008). Therefore, some measures suggested are to reduce the concentration of the active ingredient in legal pesticides and to sell products with high lethal doses (i.e. classified as acute toxicity 3 or 4 according to CLP European Regulation, Regulation (EC) No. 1272/2008, 2008) (Martínez-Haro et al., 2008). The implementation of educational programs and canine teams to look for baits and dissuade poisoners are other measures recommended (Ntemiri & Saravia, 2016; Ruiz-Suárez et al., 2015; Silva et al., 2018). In this sense, the EU Action Plan (2015) to prevent illegal poisoning of wildlife made a complete list of suggestions to improve the control over legal substances used as poison and make them less available. This included actions in marketing, national legislation, setting up a system of obligatory prescription at the point of sale and gathering detailed information in the distribution point about the amount purchased and final use of the substance and other specific information. Regarding banned products, the EU Action Plan (2015) also established strategic lines including a removal program of these substances, and an inspection, surveillance and control plan after the removal deadline is over.

Coming back to the example of ARs, these products are frequently involved in incidental cases due to a misuse or secondary poisoning, mostly not only because of their widespread use to control rodent population (Lambert et al., 2007; Ruiz-Suárez et al., 2014; Sánchez-Barbudo et al., 2012), but also because of their high persistence in organs and tissues of poisoned rodents (Gray et al., 1994). These ARs can also persist in carcasses, presenting a risk of causing tertiary poisoning (Valverde et al., 2020a). Integrated pest management (IPM) uses a combination of tools, including environmental management and physical, biological and chemical controls to reduce the use of pesticides and to monitor pest populations and development of pesticide resistance (Bajda & Grigoraki, 2020). IPM may be implemented to reduce the use of ARs to control populations of voles and rodents by combining mechanical traps and biological and chemical tools (Thomas et al., 2011). Moreover, the prohibition of chemical control in areas where biodiversity conservation is a priority over other issues should be considered (Coeurdassier et al., 2014). The addition of some repellents and the incorporation of an emetic substance in the commercial product are other measures carried out to avoid primary poisoning in non-target species (Martínez-Haro et al., 2008).

In order to support current and future regulations, it is important to carry out different toxicovigilance and risk assessment studies to reinforce the knowledge of the number of illegal poisoning cases and the substances involved in these crimes (Bille et al., 2016; Elliott et al., 2008; EU Action Plan, 2015; Mateo, 2010; Silva et al., 2018). For this purpose, many researchers and institutions/projects have suggested the creation of a network to communicate and share information between European countries about toxicovigilance, to identify each case of poisoning and to enhance the knowledge about wildlife poisoning cases (Motas-Guzmán et al., 2003; Elliott et al., 2008; Guitart et al., 2010; Mateo, 2010; EU Action Plan, 2015; Silva et al., 2018; CA16224).

The COST (European Cooperation in Science and Technology) Action *European Raptor Biomonitoring Facility* (ERBFacility; CA16224) aims to create a European network for contaminant biomonitoring in raptors (birds of prey). In this context, a Short-Term Scientific Mission (STSM) titled “Developing a Network of Analytical Labs and Government Institutions” was carried out in the National Veterinary School of Lyon (VetAgro Sup) (15 September 2019–15 December 2019). This article presents the results of the STSM aiming to create a network, focused on veterinary forensic toxicology laboratories, and to start a communication among the laboratories in the fight against wildlife poisoning, especially focused on raptors.

## Material and methods

A European network was created by first developing a questionnaire and then sending it to laboratories and institutions in Europe, and the data gathered is presented and discussed.

This entailed the creation of an email account (toxlabnetwork@hotmail.com) to communicate with the laboratories. The questionnaire was developed using SurveyMonkey® (https://www.surveymonkey.com/) as a platform.

Contact email addresses from potential laboratory candidates were obtained from different sources, including (i) Internet searching using combinations of keywords (i.e. laboratory, forensic, toxicology, wildlife, veterinary, Europe), (ii) contacting toxicology laboratories/departments in European veterinary faculties, (iii) asking for known laboratories in different European countries to the members of the ERBFacility COST Action (COST CA16224) and (iv) personal knowledge. In this sense, the list created by the EURAPMON questionnaire was used (Gómez-Ramírez et al., 2014), during the Working Group 2 Workshop on risk assessment of anticoagulant rodenticides in European raptors, held in Madrid in April 2019 (23 participants from 12 countries - Denmark, Estonia, Finland, France, Germany, Hungary, Italy, Norway, Portugal, Slovenia, Spain, the UK), and in the Working Groups 1 and 2 meeting on poisoning of raptors in Europe held in Bucharest in November 2019 (19 participants from 11 countries), participants provided additional contacts to the list.

On 17 October 2019, an email providing the link to the questionnaire was sent to 119 laboratories, but 6 could not reach the recipient due to some error in the email address. The period given to the candidates to respond the questionnaire was 3 weeks, and reminders were sent on a regular basis.

The questionnaire had a total of 39 questions grouped by different topics (i.e. laboratory information, species, wildlife species, raptors, necropsy and necropsy protocol information, analytical information, laboratory activities, legal cases, funding and other information). All questions had specific-choice answers with either one option or multiple choice. A default response “Others (Please specify)” was also provided in some questions. Moreover, according to the answers, the candidates were redirected to a different block of questions. The questions were mainly focused on wildlife and domestic animal poisoning. However, two questions were exclusively focused on raptors. The first question was: *Does your laboratory work with veterinary forensic toxicology?* If a respondent answered “No”, the questionnaire was finished and the email address was saved, and if the answer was “Yes”, the survey continued. The diagram of the questionnaire is presented in Fig. S1.

When the established response deadline arrived, the results were compiled and studied. The analysis of the data was carried out using Microsoft Excel (2016).

## Results and discussion

**Fig. 1:**
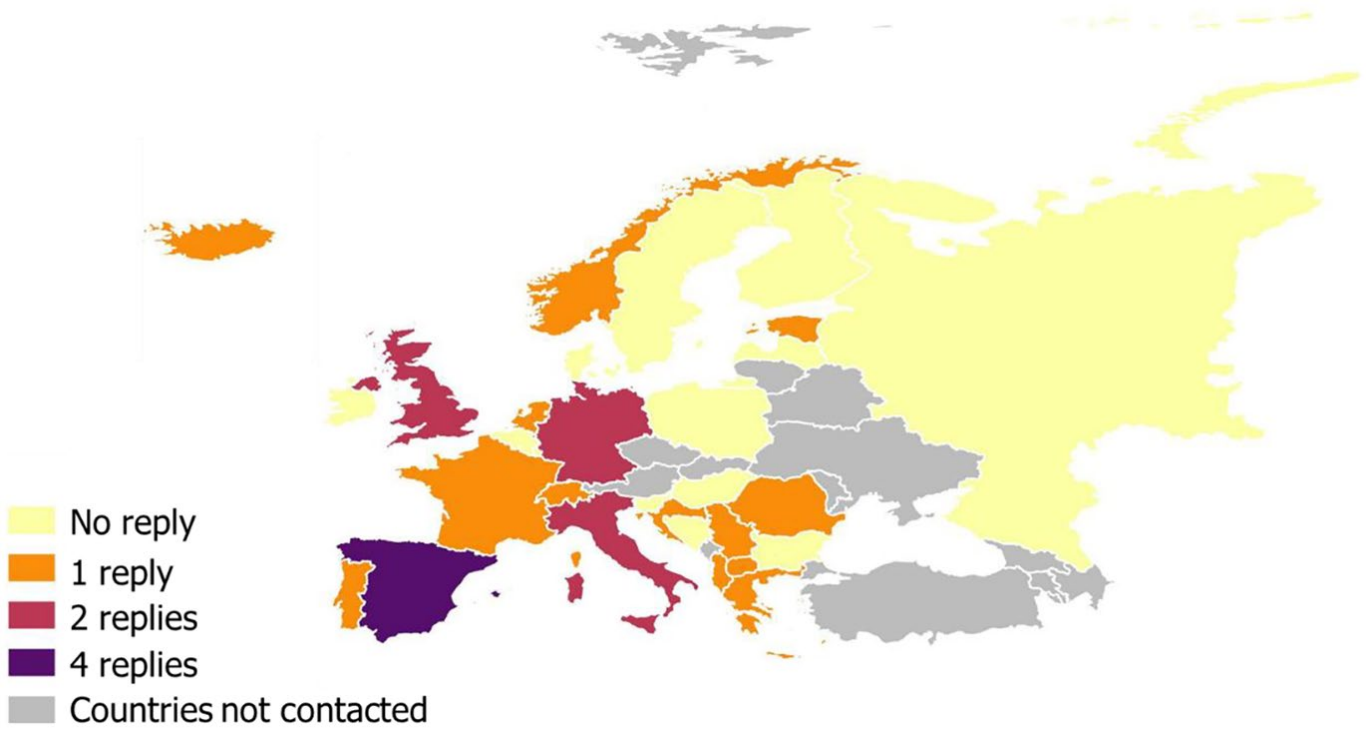
Number of replies per country laboratories contacted in Europe

The survey generated a total of 29 replies (26% response rate) (Fig. 1). However, total numbers may vary along the article because some laboratories did not reply to all the questions. From the total replies, 9 (31%) laboratories answered “No” to the first question indicating that they do not work on veterinary forensic toxicology, while 14 (48%) laboratories completed the entire questionnaire and 6 (21%) sent partially completed questionnaires. There were no responses to the questionnaire from some countries (i.e. Bulgaria, Finland, Hungary, Ireland, Latvia, Poland, Russia, Slovenia, Belgium, Luxembourg, Denmark, Bosnia and Herzegovina, and Sweden; in yellow in the map of Fig. 1). No contacts were received from the countries in grey in Fig. 1 (Austria, Belarus, Czech Republic, Lithuania, Moldova, Montenegro, Slovakia, Ukraine), and thus, we are unaware of any laboratory focused on veterinary forensic toxicology in those countries. Therefore, there is a gap of information on veterinary forensic toxicology for part of Europe (mainly northern and eastern Europe) due to incomplete questionnaires (some questions were not answered) and the lack of contacts/responses from certain countries.

**Fig. 2:**
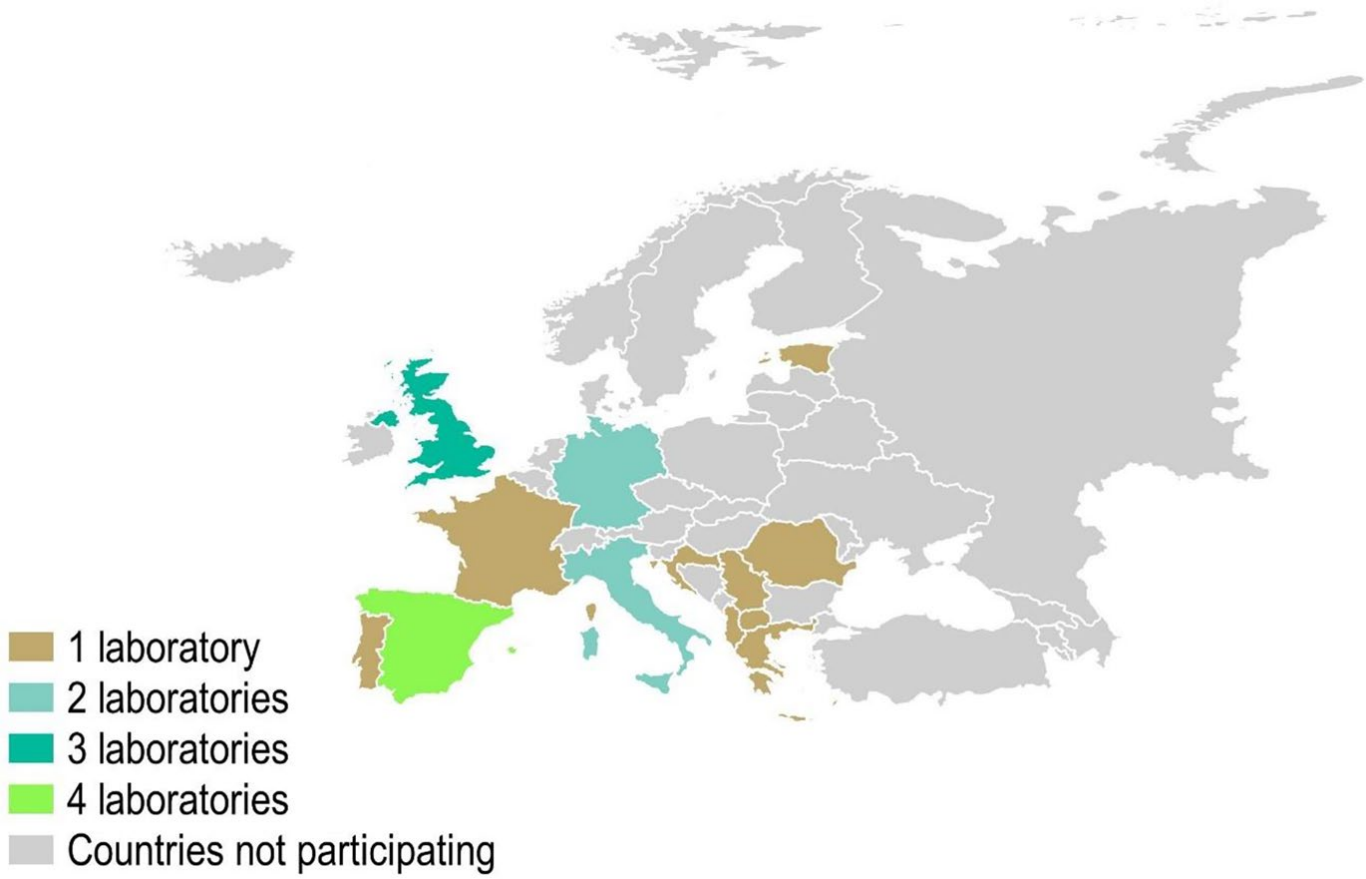
Number of laboratories in Europe working on veterinary forensic toxicology who agreed to participate in the European network (*n* = 20)

A total of 20 (69%) laboratories indicated that they work on veterinary forensic toxicology in Europe and agreed to participate in the European network. They included the following 13 countries (number of laboratories per country in brackets): Albania (1), Croatia (1), Estonia (1), France (1), Germany (2), Greece (1), Italy (2), North Macedonia (1), Portugal (1), Romania (1), Serbia (1), Spain (4), and the UK (3) (Table 1; Fig. 2). Throughout the text, we will refer to the different laboratories by their country, except for those countries with more than one laboratory. In that case, we will mention the name of the laboratory (see full laboratory names in Table 1).

**Table 1:** List of laboratories in Europe working on veterinary forensic toxicology who agreed to participate in the European network (*n* = 20)

Country	Lab name
Albania	Centre for Wildlife Investigation and Health, Faculty of Veterinary Medicine, Agricultural University of Tirana (CWIH)*
Croatia	Laboratory of Pathology, Croatian Veterinary Institute, Poultry Centre (LP)*
Estonia	Estonian University of Life Sciences, Institute of Veterinary Medicine and Animal Sciences (EULS)*
France	Toxicology Laboratory (ToxLab), Vetagro Sup, Veterinary Campus
Germany	Leibniz Institute for Zoo and Wildlife Research, Department of Wildlife Diseases, Toxicological laboratory (LIZW)*
Germany	Ludwig-Maximilians-University of Munich, Faculty of Veterinary Medicine, Institute of Pharmacology, Toxicology and Pharmacy (LMUM)*
Greece	Toxicology lab, Department of toxicology, residues and environmental contaminants, Ministry of Development and Food (TL)*
Italy	Centro di Referenza Nazionale per la Medicina Forense Veterinaria Istituto Zooprofilattico Sperimentale delle Regioni Lazio e Toscana "M. Aleandri" (CRNMFV)*
Italy	Istituto Zooprofilattico Sperimentale delle Venezie (IZSVe)
Macedonia	Faculty of Veterinary Medicine Skopje (FVMS)*
Portugal	Laboratório de Histologia e Anatomia Patológica da Universidade de Trás-os-Montes e Alto Douro (LHAP)*
Romania	Animal Behaviour and Ecotoxicology research group (ABERG)*
Serbia	Department of Drug Analysis and Veterinary Toxicology, Scientific Veterinary Institute Novi Sad, Novi Sad (DDAVT)*
Spain	Instituto de Investigación en Recursos Cinegéticos (IREC)
Spain	Service of Toxicology and Forensic Veterinary, University of Murcia (STVF)
Spain	Servicio de Toxicología Clínica y Analítica (SERTOX), University of Las Palmas de Gran Canaria
Spain	Veterinary Analytical Toxicology Laboratory, University of Extremadura (VATL)*
UK	Agri-food and Biosciences Institute (AFBI)
UK	Fera Science Ltd (Fera)
UK	Science & Advice for Scottish Agriculture (SASA)

*Acronyms have been created when they were not provided.

Among the laboratories working with veterinary forensic toxicology, 15 (75%) work on both research and external cases (2 from Italy, 4 from Spain, 2 from the UK (Fera and SASA) and 1 from Germany (LIZW) and the laboratories from Portugal, France, Serbia, Albania, Estonia and Croatia). Three (15%) laboratories only work with external cases (Germany (LMUM), Greece and the UK (AFBI)), while 2 (10%) laboratories carry out only research work (Romania and Macedonia). Sixteen (80%) laboratories work with domestic animals and wildlife samples, whereas 4 (20%) laboratories only work with wildlife samples (Germany (LIZW), Romania, Albania and Estonia).

### Domestic animals, wildlife groups and raptor species

**Fig. 3:**
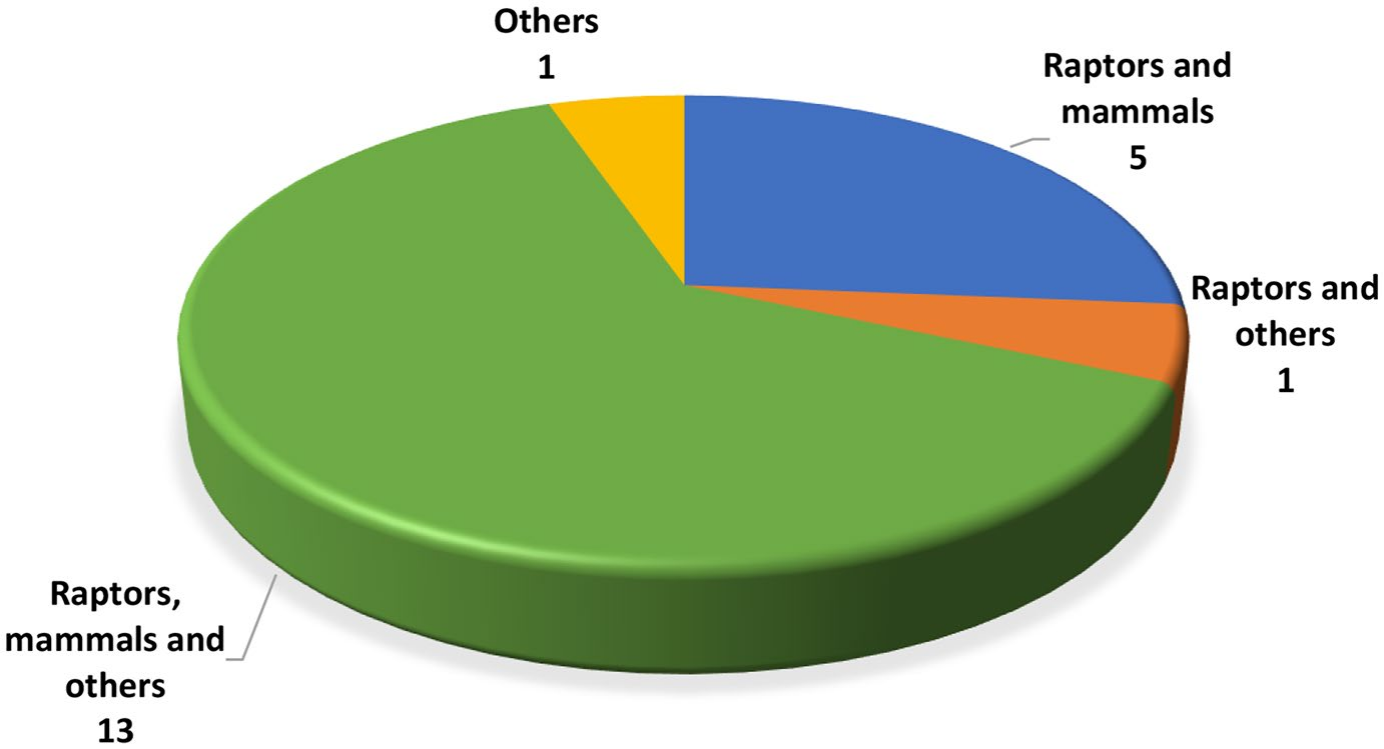
Wildlife species analysed in participant laboratories

Regarding wildlife, 19 (95%) laboratories receive samples from raptors and other animal groups such as other birds, reptiles, fish, bees and mammals (Fig. 3). Many also receive baits for analysis. The laboratory from Romania is the only one that does not work with raptor samples, but it receives samples that include other birds, fish and aquatic invertebrates.

Fourteen of the most common raptor species in Europe were listed in the questionnaire to estimate the number of specimens received per species and year by each laboratory (Table 2). Common buzzard (*Buteo buteo*) (*n* = 16, 94% laboratories), Eurasian sparrowhawk (*Accipiter nisus*) (*n* = 13, 76%), red kite (*Milvus milvus*) (*n* = 12, 71%), golden eagle (*Aquila chrysaetos*) (*n* = 12, 71%) and northern goshawk (*Accipiter gentilis*) (*n* = 12, 71%) were the raptor species most frequently received (*n* = 17). The little owl (*Athene noctua*) is the raptor species least commonly received (*n* = 7, 41%). The highest frequency of the common buzzard (all laboratories except for North Macedonia) is probably due to its widespread distribution in the western palaearctic. Moreover, this species is an active hunter and a facultative scavenger, which makes it susceptible to exposure not only to contaminants accumulated in the trophic chain, but also to primary and secondary poisoning (e.g. ARs or lead (Pb) from ammunition sources). In fact, due to both its distribution and diet, the common buzzard has been suggested as a good key species in pan-European biomonitoring studies (Badry et al., 2020; Schindler et al., 2012).

**Table 2:** Number of laboratories in Europe receiving raptor species and number of individuals received per year (*n* = 17)

Species	Not received	Received	Individuals/year			
			< 5	5–20	20–35	> 35
*Buteo buteo*	1	16	3	7	4	2
*Accipiter nisus*	3	13	10	3	0	0
*Accipiter gentilis*	5	12	9	3	0	0
*Aquila chrysaetos*	5	10	8	2	0	0
*Milvus milvus*	2	12	5	5	2	0
*Falco peregrinus*	5	11	8	3	0	0
*Falco tinnunculus*	4	11	8	2	1	0
*Tyto alba*	5	11	7	4	0	0
*Bubo bubo*	6	10	9	0	0	1
*Gyps fulvus*	6	9	2	4	1	2
*Strix aluco*	4	10	7	3	0	0
*Circus pygargus*	6	9	9	0	0	0
*Milvus migrans*	6	9	6	2	1	0
*Athene noctua*	9	7	6	1	0	0

### Compounds analysed in poisoning investigation

**Fig. 4:**
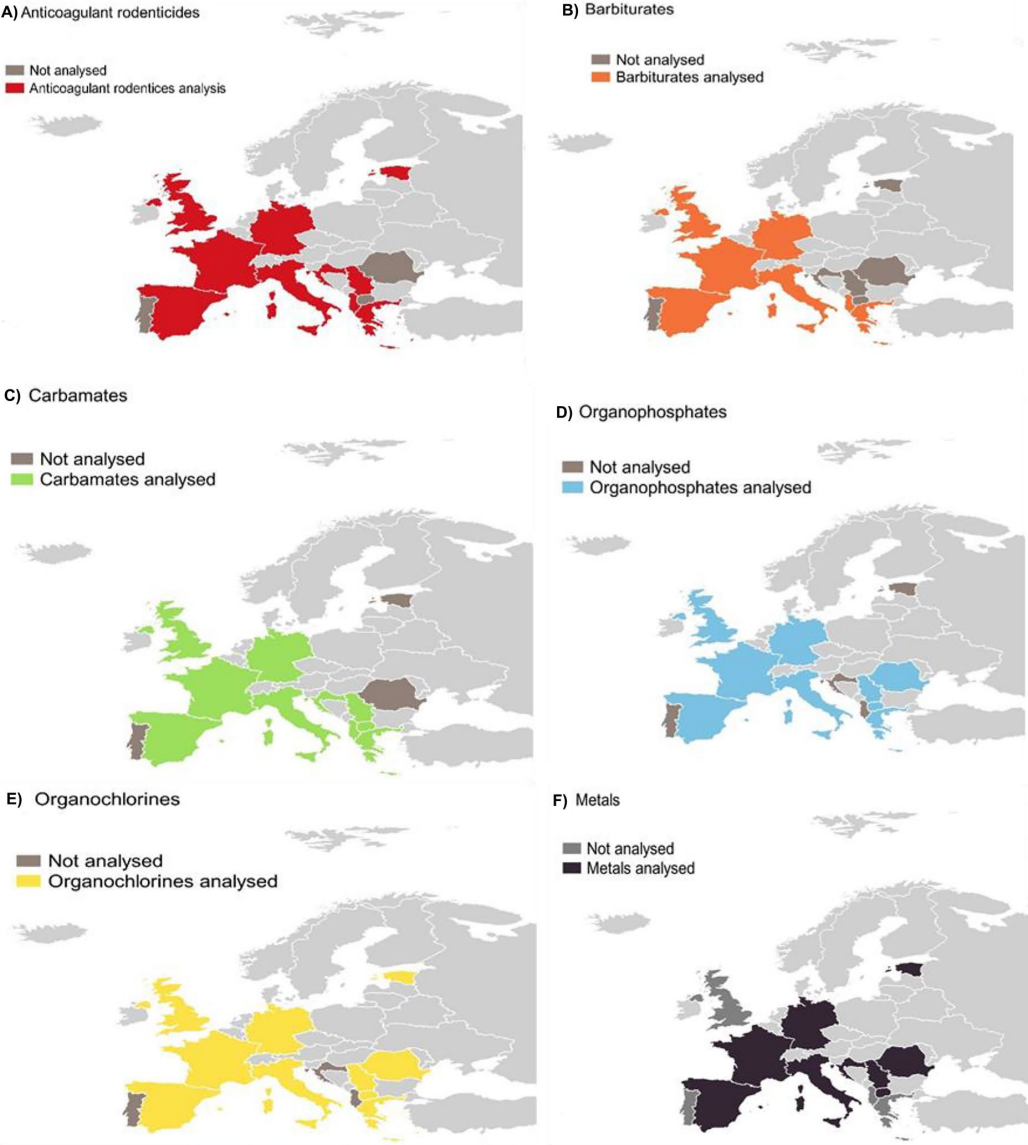
Categories of compounds analysed in each country

The groups of most analysed compounds by the participant laboratories are ARs (*n* = 15, 83%), carbamates (*n* = 15, 83%) and organochlorines (OCs) (*n* = 15, 83%). Figure 4 represents the distribution of compounds analysed per country. Regarding detection in raptor poisoning cases in the European laboratories, carbamates, ARs and organophosphates (OPs) were the group of compounds most frequently detected. This is consistent with the literature on poisoning cases (Berny et al., 2010; Chiari et al., 2017; Grilo et al., 2021; Guitart et al., 2010; Motas-Guzmán et al., 2003; Parvanov et al., 2018; Uros & Andevski, 2018).

Barbiturates are pharmaceuticals widely used in veterinary medicine, and they are involved in accidental secondary poisoning (Herrero-Villar et al., 2021; Wells et al., 2020). Nevertheless, our results showed that few laboratories analysed them in Europe (*n* = 8, 44%), being the group of compounds less frequently analysed (Fig. 4). Other compounds analysed but in fewer laboratories (*n* = 8, 44%) were neonicotinoids (imidacloprid), pyrethroids, deltamethrin, other veterinary pharmaceuticals (antibiotics, non-steroidal anti-inflammatory drugs (NSAIDs), hormones, paracetamol, benzodiazepines, levamisole, etc.), pyrogallol, colchicine, phosphine, cyanides, brucine and ethylene glycol.

Some compounds such as glyphosate and ethylene glycol require further attention since they are rarely mentioned in the literature (Berny et al., 2010; Modrä & Svobodová, 2009; Uros & Andevski, 2018). Only two (11%) laboratories analyse glyphosate (IREC from Spain and Fera from the UK) and the CRNMFV laboratory from Italy analyses ethylene glycol.

According to poisoning reports, most laboratories have developed techniques to detect the most frequently used compounds to poison animals. Table 3 shows the matrices and the analytical methods used to analyse each compound group in the 19 European laboratories that responded to this section of the questionnaire. Fig. S2 represents the specific compounds analysed within each compound group in the different laboratories.

**Table 3:** Compound groups by matrix and analytical methods used to analyse each compound group

		ARs^a^	Barbiturates	Carbamates	Pharmaceuticals	Metals	Metaldehyde	OCs^a^	OPs^a^	Strychnine	α-Chloralose	Other
**Matrix (n = 15)**	Blood	7	5	6	5	11	3	5	5	4	4	5
	Plasma	4	3	4	6	4	3	4	4	3	3	3
	Gastric content	6	10	15	9	7	12	10	14	10	9	8
	Kidney	5	3	5	5	11	3	11	9	7	6	8
	Liver	12	7	12	7	12	3	11	10	7	6	8
	Baits	11	8	14	8	8	11	11	13	10	10	7
**Methods (n = 14)**	HPLC^b^-UV^b^/DAD^b^/Fluo^b^	5	0	1	NR	NA	0	0	0	1	0	NR
	LC–MS-MS^b^	7	1	9	NR	NA	3	1	4	6	4	NR
	GC^b^	0	0	0	NR	NA	0	2	0	0	1	NR
	GC–MS	1	8	5	NR	NA	6	11	11	6	5	NR
	AAS^b^	NA	NA	NA	NR	7	NA	NA	0	NA	NA	NR
	ICP^b^/ICP-MS	NA	NA	NA	NR	7	NA	NA	0	NA	NA	NR
	Other	2	0	1	NR	2	1	0	0	0	0	NR

*NA* not applicable, *NR* This information was not required in the questionnaire.

^a^ARs, anticoagulant rodenticides; OCs, organochlorines; OPs, organophosphates.

^b^AAS, atomic absorption spectroscopy; DAD, diode-array detector; Fluo, fluorescence; GC, gas chromatography; HPLC, high-performance liquid chromatography; ICP, inductively coupled plasma; MS, mass spectrometry detector; UV, ultraviolet detector.

### Matrices analysed in poisoning investigation

Baits, gastric content and liver were the most frequently used matrices to analyse poisoning substances among the respondents. These three matrices are preferred sample types for the detection of common substances (Berny, 2007), since they are linked with oral exposure, which is the most common route of exposure for animals (Giorgi & Mengozzi, 2011; Mineau & Tucker, 2002). After ingestion, the substances are absorbed and distributed through the body via the blood, where they usually remain for a short time. Concentrations in blood represent a recent exposure. However, in starving or migrating birds, a redistribution of substances may be possible. Thus, blood is a useful sample in live animals, while it is not such a good matrix in dead animals (Espín et al., 2016; Mateo et al., 2013). In addition, the liver is the principal metabolizing (Watt et al., 2005) and, in many cases, accumulating organ, which will allow us to confirm that the substance has been absorbed, mainly from the ingesta (Thomas, 1999), but also after dermal or respiratory exposure. Concentrations in tissues, like the liver, not only determine medium or long-term exposure of cumulative compounds, but can also help to monitor recent exposure to many contaminants, including pesticides (Espín et al., 2016). Although the choice of the target matrix should be determined by the toxicokinetics and toxicodynamics of the substances (García-Fernández, 2014), tissues which accumulate the highest contaminant concentrations are sometimes analysed in reference to the target organ (Espín et al., 2016).

In poisoning cases, the substances most commonly involved are carbamates and OPs, which are quickly metabolized in the body, so the use of gastric content and liver as target sample is useful (Mateo et al., 2013). In addition, baits help identify the compound involved during analysis because it is likely to be found at high concentrations in this sample (Mateo et al., 2013; Motas-Guzmán et al., 2003). Visual inspection of the gastric content can assist in detection of compounds before analysis (e.g. by the presence of granulated material or coloured content) and help link a bait to a poisoning (Cenerini et al., 2012).

**Fig. 5:**
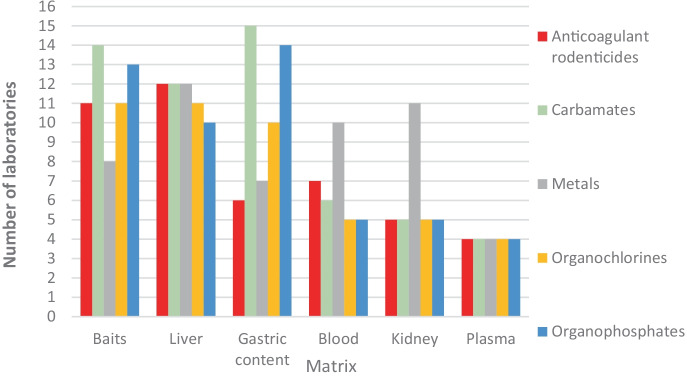
Matrices used in veterinary toxicology to analyse the principal groups of compounds in poisoning cases (*n* = 18 laboratories)

In contrast, plasma and kidney are not very often analysed (Fig. 5). Plasma, like blood, cannot be obtained from dead animals in most intances, so it is only used for diagnostic purposes in live animals. Nevertheless, plasma concentrations of some compounds like ARs can be good predictors of clinical poisoning of raptors (Murray, 2020). Although we have collected information about the main samples used to diagnose poisoning, sometimes less suitable samples are available because of the state of decomposition of carcasses. As an example, Martínez-López et al. (2006) found strychnine in fragments of the remaining tissue adhering to the vertebral column and ribs, from the area corresponding to the anatomical location of the liver and stomach.

In summary, matrices and analytical techniques to analyse the same compounds are diverse. An important methodological issue would be to harmonise analytical methods across European laboratories during the creation of the network, in order to improve the homogeneity of results and also develop common interpretation strategies, based on comparable results.

### Matrices and methods used to analyse each group of compounds

As evidenced in the literature, and due to their chemical and pharmaceutical properties (Espín et al., 2016; Valverde et al., 2021), ARs were found to be mostly analysed in liver and baits by LC–MS-MS, except in the laboratory from Greece, where they are analysed with GC–MS technique (Table 3). Among them, bromadiolone (93%) and brodifacoum (87%) were the most frequently analysed (Fig. S2), probably because they are frequently found in wildlife poisoning and widely used to control rodent pests (Berny & Gaillet, 2008; Langford et al., 2013; Valverde et al., 2021).

According to the bibliography (Espín et al., 2016) and to this survey, the most common matrices to analyse carbamates, OPs, metaldehyde, strychnine and α-chloralose are gastric content and baits, followed by the liver.

Carbamates are mostly analysed by LC–MS, while OPs and metaldehyde are mainly analysed by GC–MS, and both instruments are similarly used for strychnine and α-chloralose (Table 3). Carbofuran (100%) is the carbamate most frequently analysed, while chlorpyrifos (100%) and diazinon (93%) are the OPs most frequently analysed (Fig. S2). Carbofuran, together with aldicarb, are by far the carbamates most frequently involved in poisoning cases (Guitart et al., 2010; Modrä & Svobodová, 2009; Ntemiri & Saravia, 2016; Ruiz-Suárez et al., 2015), despite the fact that both were banned in 2008 and 2007, respectively (Decision 2003/199/EC, 2003; Decision 2007/416/EC, 2007). Chlorpyrifos has been recently banned (Commision Regulation (EU) 2020/1085 2020), and diazinon was banned in 2007 (Decision 2007/393/EC, 2007); nevertheless, both OPs are still involved in poisoning cases (Ntemiri & Saravia, 2016; Ruiz-Suárez et al., 2015). On the contrary, diazinon is rarely detected.

Organochlorines in the liver and baits are more frequently analysed by GC–MS, except in the laboratory from Serbia, where they are analysed by LC–MS (Table 3). Lindane (87%) and endosulfan (80%) are the OCs most frequently analysed (Fig. S2) and also the most detected in cases of poisoning (Bertero et al., 2020; Hernández & Margalida, 2009; Martínez-Haro et al., 2008). Organochlorines are usually analysed not only in the liver, fat and brain, but also in stomach content and plasma (Berny, 2007; Espín et al., 2016). In the case of metals, the most frequently used matrices are the liver, kidney and blood. In general, they are analysed by ICP/ICP-MS or AAS (Table 3). Lead (100%) is the most analysed metal (Fig. S2). According to the review by Espín et al. (2016), the liver and kidney are the most used tissues to analyse metals, and blood is mainly used to detect high levels of Pb. Although, normally animals are unintentionally poisoned by Pb, it is a metal of concern in hunting activities, since birds, mainly scavengers and waterfowl, are highly exposed to the ingestion of Pb ammunition (Mateo et al., 1997; Garcia-Fernandez et al., 2005; Guitart et al., 2010; Espín et al., 2014; Berny et al., 2015).

The matrices used to analyse pharmaceuticals are diverse (Espín et al., 2016), since this group includes many different substances of different classes (e.g. antibiotics, NSAIDs, hormones, benzodiazepines, antiparasitics). Barbiturates are always analysed in gastric content and baits, followed by the liver and blood. They are analysed with GC–MS, except in the laboratory SERTOX from Spain, where they are analysed with LC–MS (Table 3). Pentobarbital is the most commonly analysed compound within this group (100%) (Fig. S2). This is the most used pharmaceutical to euthanize domestic animals that may be eaten by scavengers and become a secondary-poisoning source (Herrero-Villar et al., 2021; Wells et al., 2020). Pentobarbital is well detected in gastric content and the liver (Friend & Franson, 1999).

### Necropsy

Necropsies are an important step in the study of poisoning cases since they provide much information before the laboratory analysis (Valverde et al., 2020a, 2020b). Table S1 compiles information about necropsy questions. In those laboratories that perform necropsies (*n* = 12, 63%), the main points of focus are the anamnesis history, the presence of haemorrhages, the nature of gastric content and the presence of other lesions. In 5 laboratories (Portugal, Serbia, Croatia, IZSVe (Italy) and STVF from Spain), more than 100 necropsies per year are performed. Five laboratories provide specific necropsy veterinary forensic training to their staff (LIZW from Germany, Portugal, Estonia and STVF from Spain) (Table S1), and 10 (83%) laboratories have a necropsy protocol. Three (30%) of them never carry out X-ray (Serbia, Albania and North Macedonia), four (40%) laboratories always do X-ray because it is part of their protocol (LIZW from Germany, Portugal, Estonia and IREC from Spain) and three (30%) laboratories do X-ray when a trauma is suspected (STVF from Spain and 2 laboratories from Italy) (Table S1). Nine (90%) laboratories estimate the date of death, and most of them use the overall status and forensic entomology, but the laboratory in Estonia uses all relevant findings in combination of weather and species biology (Table S1).

The information obtained during necropsies is essential to better investigate suspected poisoning cases before performing analytical procedures (Brown et al., 2005; Mateo et al., 2013; Valverde et al., 2020b). Proper protocols for collecting information and contextual data in the field and during necropsy, as well as proper sample collection and estimation of carcass decomposition and time of death, are essential for a successful resolution of poisoning cases (Espín et al., 2021; Mateo et al., 2013; Valverde et al., 2020b). These are important issues that should be considered to harmonise practices in the future.

### Funding and costs

The average cost of toxicological analysis ranges from 50 to 250 € per sample, and funding is mostly provided by the governments (Table S2). In the majority of laboratories (79%), funding comes from the government, also combined with (non-governmental organizations) NGOs and private sources. A notable exception is the UK: the two laboratories participating in the survey only receive funding from the government. For the laboratoy from Romania, funding comes from research projects, and the laboratory from Estonia also receives funding on a “project basis” or “through universities internal resources”.

Table S2 details the costs of analysis and the number of compounds analysed, the use of reference material and the accredited labs. In general, laboratories with lower price per analysis (< 50 €) do not analyse more than 18 compounds, while laboratories with higher prices analyse a larger number of compounds. This may be explained because the development of new analytical techniques implies higher laboratory costs. Moreover, laboratories with prices of 50–250 or > 250 € always provide interpretation of the results and legal reports, which also implies more workload, time and experienced personnel.

The origin of the funding may also determine the prices offered by the laboratories. The funding of the laboratories whose prices range between 50 and 250 €, in most cases, comes from the government; nevertheless, laboratories with < 50 € prices are those with private or NGOs financial support.

### Other information

Different questions about other laboratory routines were also asked in the survey (Tables S3 and S4). Ten laboratories (59%) provide toxicology training to their staff. Most of them (14 laboratories, 78%) are able to process samples from outside of the institution and/or cooperate with other countries. Laboratories from Romania, UK, Italy and Croatia publish data online (see Table S4).

In general, laboratories report results within 15–30 days. Regarding the use of reference materials, most laboratories (83%) use at least some analytical reference material, while three (17%) of them do not have them available. Regarding the laboratories with accreditation (2 from the UK and Italy, Serbia and North Macedonia), all of them have quality assurance ISO 17025 (Table S3). Most laboratories (15 laboratories, 83%) provide interpretation of the analytical results (Table S4), which may be a helpful step considering that many clients are not specialists in toxicology.

**Fig. 6:**
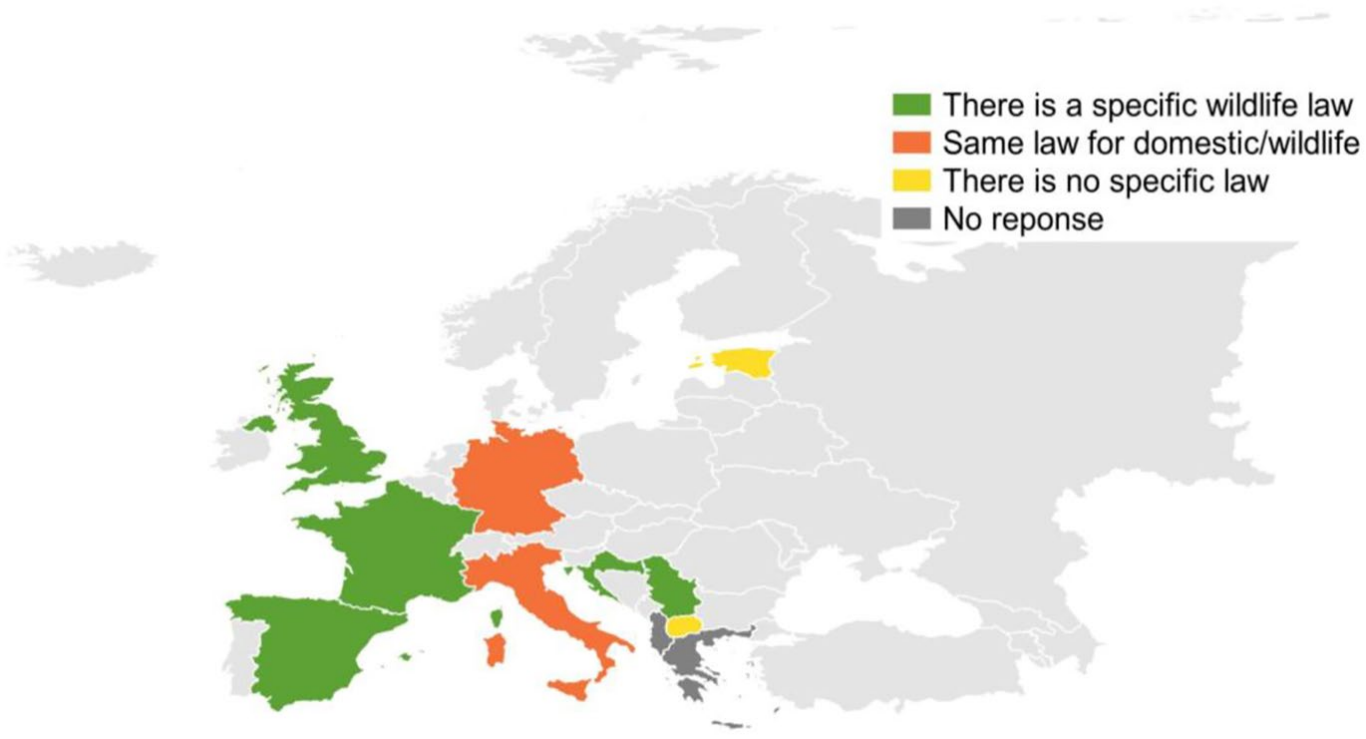
Wildlife legislation

With respect to legal cases (Table S5), 15 laboratories (83%) prepare legal reports. The legislation on animal poisoning in European countries is extensive. There is international legislation such as conventions and treaties (Directive 92/43, 1992; Directive 2008/99/EC, 2008; Directive 2009/147/EC, 2010), and there are laws or regulations in each country regulating specifically wildlife poisoning (Bille et al., 2016; Ntemiri & Saravia, 2016). However, to our knowledge, North Macedonia does not have a specific law for intentional animal poisoning. Germany and Italy have the same law for domestic animals and wildlife, and in the UK, France, Serbia, Croatia and Spain, there is a specific wildlife law. In Estonia, there is no specific law, but it is regulated through multiple legislation indirectly; however, some are straighter forward (Fig. 6).

Albania, Romania and Greece did not answer this part of the questionnaire. However, the questionnaire was not specifically designed to investigate this point and did not provide enough information to go in depth into the legislation and its efficacy in each country.

The creation of a coordinated European network may help countries to keep updated regarding the current products used to poison animals, spatiotemporal differences in their use (e.g. metaldehyde baits are more often reported in southern Italy (De Roma et al., 2018), whereas insecticides and ARs are more frequent in northern Italy (Chiari et al., 2017; Giorgi & Mengozzi, 2011).

Finally, in order to avoid a lengthy and tedious questionnaire, technical questions related to quantity of sample, extraction procedure, limits of quantification and additional questions about necropsy findings were not asked. For this reason, a new survey is recommended to obtain detailed information on these issues.

## Conclusions

Unfortunately, illegal wildlife poisoning is a frequent occurrence in Europe. To evaluate and prevent such acts, a fluent communication and coordination among laboratories in Europe is needed. Therefore, the present study represents a first contact among European laboratories as an initial step to create a network and compile basic data from a survey to detect strengths and pitfalls that will help to harmonise methodologies and increase pan-European capacities.

Most laboratories, participating in the present study, work on veterinary forensic toxicology research and external cases at the same time, which can give a broad overview of the actual situation in the field.

Various analytical techniques, sample requirements and data collection techniques should be harmonised, and a sufficient communication among laboratories is needed to create an effective network. All respondents reacted positively to this suggestion.

To continue the network development, the following guidelines need to be considered:

(i) An online platform should be created, with free access to detailed information on each laboratory (e.g. contact, address, analytical techniques available, prices). Data on poisoning cases should be uploaded/updated regularly by each laboratory. Such data should contain information regarding the location where the sample was collected, species, type of samples and analytical techniques used, detected compound/s and basic necropsy information (if it is accessible). A simple online necropsy protocol could be developed for this purpose to identify the principal necropsy findings (see some suggestions at Mateo et al., 2013; Valverde et al., 2020b). Furthermore, an online forum could be developed to share opinions and seek for assistance in complex cases or for technical purposes to other colleagues in the network.

(ii) Laboratories should analyse, at least, carbamates, organophosphates and anticoagulant rodenticides in suspected poisoning cases using the liver, baits and/or gastric content as key samples. If this is not possible, laboratories could contact others in the network to send the samples and perform the analyses.

(iii) The compilation of clear protocols describing how to collect, pack and send samples to other laboratories should be carried out.

(iv) A new survey to obtain additional information about sample quantity, extraction and analytical techniques is needed to improve and harmonise methodologies in Europe.

(v) Common analytical work to validate new wildlife forensic toxicology analytical procedures including non-invasive samples such as feathers and hair is needed.

All the information gathered in the present study as well as the recommendations provided are a first step to develop a pan-European network of analytical laboratories and government institutions to fight against wildlife poisoning.

## Supplementary Information

Below is the link to the electronic supplementary material.

Supplementary file1 (DOCX 113 KB)

## Data Availability

Data will be available upon request contacting irene.valverde@um.es and silvia.espin@um.es.
